# Effect of Low-Carbohydrate Diets on Glycemic Control in Type 2 Diabetes Mellitus: A Systematic Review and Meta-Analysis of Randomized Controlled Trials

**DOI:** 10.7759/cureus.108479

**Published:** 2026-05-08

**Authors:** Yazan Khraise

**Affiliations:** 1 Internal Medicine, Jordan University of Science and Technology, Irbid, JOR

**Keywords:** dietary intervention, glycemic control, hba1c, insulin resistance, ketogenic diet, low-carbohydrate diet, meta-analysis, randomized controlled trials, type 2 diabetes

## Abstract

Low-carbohydrate diets have gained increasing attention as a strategy for improving glycemic control in individuals with type 2 diabetes mellitus (T2DM). However, evidence from randomized controlled trials (RCTs) remains variable. This study aimed to evaluate the effect of low-carbohydrate diets on HbA1c levels in patients with T2DM through a systematic review and meta-analysis of RCTs.

A systematic search was conducted to identify RCTs comparing low-carbohydrate diets with control diets in adults with T2DM, including studies with mixed populations where applicable. The primary outcome was change in HbA1c. Data were pooled using a random-effects model, and heterogeneity was assessed using the I² statistic. Sensitivity analysis and funnel plot assessment were performed. Risk of bias was assessed using the Cochrane Risk of Bias 2 tool.

Seven RCTs comprising 562 participants were included. Low-carbohydrate diets were associated with a statistically significant reduction in HbA1c compared with control diets (mean difference: -0.24%; 95% CI: -0.32 to -0.16; p<0.00001). Heterogeneity was low (I²=6%). Sensitivity analysis demonstrated consistent results with no single study significantly influencing the overall estimate. Funnel plot assessment showed no clear evidence of publication bias, although interpretation was limited by the small number of studies.

Low-carbohydrate diets are associated with a modest but statistically significant and clinically relevant improvement in glycemic control in patients with T2DM. These findings support their role as a dietary strategy in diabetes management.

## Introduction and background

Type 2 diabetes mellitus (T2DM) is a major global health concern affecting hundreds of millions of individuals worldwide and characterized by chronic hyperglycemia and associated with significant morbidity and mortality [1]. Effective glycemic control is essential to prevent microvascular and macrovascular complications [2].

Dietary modification remains a cornerstone in the management of T2DM [1,3]. Among various dietary approaches, low-carbohydrate diets have gained popularity due to their potential to improve glycemic control by reducing postprandial glucose excursions and insulin demand [4,5].

Low-carbohydrate diets are generally defined as dietary patterns that restrict carbohydrate intake to less than 130 g per day, with very-low-carbohydrate or ketogenic diets typically limiting intake to less than 50 g per day [6].

Despite increasing interest, the evidence regarding the effectiveness of low-carbohydrate diets remains inconsistent across randomized controlled trials (RCTs) [5,7], with reported reductions of HbA1c generally being modest and typically less than 1 percentage point. Differences in study design, carbohydrate thresholds, and duration of intervention contribute to variability in reported outcomes.

This systematic review and meta-analysis aim to synthesize current evidence from RCTs to determine the effect of low-carbohydrate diets on HbA1c in individuals with T2DM.

Existing evidence remains heterogeneous and sometimes conflicting, despite increasing interest in low-carbohydrate dietary approaches.

## Review

Methods

Study Design

This study was conducted as a systematic review and meta-analysis of RCTs.

Search Strategy

A systematic literature search was conducted in PubMed (MEDLINE) and the Cochrane Library from database inception to March 2026. The search strategy combined terms related to low-carbohydrate and ketogenic diets, type 2 diabetes mellitus, and glycated hemoglobin (HbA1c) using Boolean operators. Full database-specific search strategies are provided in the Appendices. Google Scholar was used as a supplementary source to identify potentially relevant studies; however, due to its limited reproducibility, results were not included in the formal screening process. Reference lists of relevant articles and systematic reviews were also hand-searched to identify additional eligible studies.

Study Selection

The study selection process was conducted in accordance with the Preferred Reporting Items for Systematic Reviews and Meta-Analyses (PRISMA) guidelines. All records identified through database searching were imported, and duplicate records were identified and removed prior to screening. Due to the single-author nature of this systematic review and meta-analysis, titles and abstracts of all retrieved records were screened by the author (YK). Full-text articles deemed potentially eligible were retrieved and assessed for inclusion against the pre-specified eligibility criteria. Uncertain cases were re-evaluated to ensure consistency in study selection.

Eligibility Criteria

The eligibility criteria for study inclusion were defined according to the PICO framework. The population (P) included adults with T2DM or mixed populations including individuals with prediabetes. The intervention (I) consisted of low-carbohydrate or ketogenic diets. The comparison (C) included standard diets, higher-carbohydrate diets, or usual care. The primary outcome (O) was change in HbA1c levels.

Studies were included if they were RCTs evaluating low-carbohydrate or ketogenic diets in adults with T2DM or mixed populations with elevated HbA1c (including prediabetes) and reporting HbA1c outcomes. Studies were excluded if they were observational studies, reviews, editorials, or case reports, involved normoglycemic populations without diabetes or prediabetes, or did not report HbA1c.

Data Extraction

Data extraction was performed by the single author using a standardized, piloted data extraction form. Extracted data included study characteristics (author and year), sample size, participant demographics, intervention and comparator details, duration of intervention, and HbA1c outcomes, including mean change and standard deviation (SD). To minimize errors, all extracted data were independently verified through repeated review.

Statistical Analysis

Meta-analysis was performed using a random-effects model to account for between-study variability. Results were expressed as mean difference (MD) with 95% confidence intervals (CI). Statistical analyses were conducted using Review Manager (RevMan, Version 5.4, The Cochrane Collaboration, London, England, United Kingdom).

A random-effects model was used to account for expected variability between studies, including differences in dietary interventions, participant characteristics, and follow-up durations.

Heterogeneity was assessed using the chi-squared test and I² statistic.

Sensitivity Analysis

Sensitivity analysis was conducted by sequentially removing individual studies to assess the robustness of the pooled estimate.

Publication Bias

A funnel plot was generated to assess publication bias. However, due to the small number of included studies (<10), interpretation was considered limited.

Risk of Bias Assessment

Risk of bias was assessed using the Cochrane Risk of Bias 2 (RoB 2) tool for RCTs. Each included study was evaluated across five domains: bias arising from the randomization process, bias due to deviations from intended interventions, bias due to missing outcome data, bias in measurement of the outcome, and bias in selection of the reported result. Each domain was classified as low risk, some concerns, or high risk of bias, and an overall risk of bias judgment was assigned for each study.

Protocol and Registration

This systematic review and meta-analysis was not prospectively registered in the International Prospective Register of Systematic Reviews (PROSPERO) or another protocol registry.

Results

Study Selection

**Figure 1:**
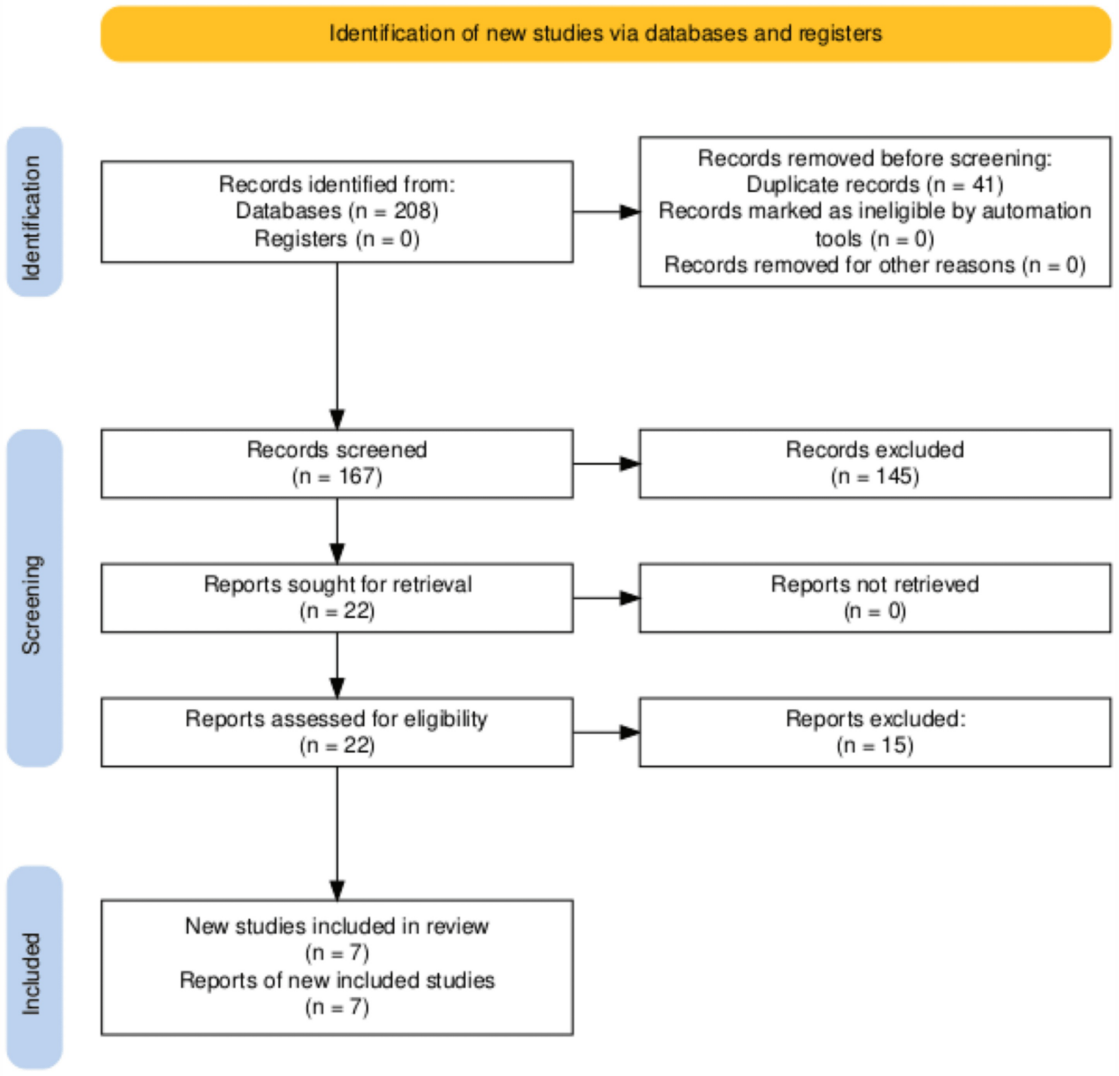
PRISMA flow diagram of study selection PRISMA: Preferred Reporting Items for Systematic Reviews and Meta-Analyses

A systematic search of databases yielded 47 records from PubMed and 161 from the Cochrane Library, for a total of 208 records. Google Scholar was used for supplementary screening of potentially relevant studies; however, due to its non-reproducible nature, results were not formally included in the PRISMA flow diagram. After the removal of 41 duplicate records, 167 records were screened, of which 145 were excluded based on title and abstract screening. A total of 22 full-text articles were assessed for eligibility, with no reports unavailable for retrieval. Fifteen full-text articles were excluded for the following reasons: non-randomized study design (n=8), lack of an appropriate control group (n=4), and insufficient HbA1c outcome data (n=3). Hand-searching of reference lists did not identify any additional eligible studies. Ultimately, seven RCTs [8-14] were included in the meta-analysis (Figure 1).

Study Characteristics

A total of 562 participants were included in the pooled analysis (282 in the intervention group and 280 in the control group). All studies evaluated the effect of low-carbohydrate or ketogenic diets on glycemic control in individuals with T2DM or related metabolic conditions. The characteristics of the included studies are summarized in Table 1.

**Table 1:** Characteristics of the included randomized controlled trials evaluating low-carbohydrate diets and HbA1c outcomes "Create Your Plate" diet is an American Diabetes Association plate-method dietary approach in which half of a nine-inch plate is filled with non-starchy vegetables, one-quarter with carbohydrates, and one-quarter with lean protein. DASH: Dietary Approaches to Stop Hypertension

Study	Year	Country	n	Intervention	Control	Duration	Carb intake	Outcome
Saslow et al. [8]	2017	USA	25	Very-low-carbohydrate ketogenic diet	"Create your plate" diet	8 months	20-50 g/day	HbA1c reduction
Saslow et al. [9]	2023	USA	94	Very-low-carbohydrate diet+behavioral support	DASH diet	4 months	20-35 g/day	HbA1c reduction
Dorans et al. [10]	2022	USA	150	Low-carbohydrate diet	Usual diet	6 months	<40 g/day (months 1-3); <60 g/day (months 4-6)	HbA1c reduction
Chen et al. [11]	2020	China	92	Low-carbohydrate diet	Traditional diabetic diet	18 months	<90 g/day	HbA1c reduction
Thomsen et al. [12]	2022	Denmark	72	Carbohydrate-reduced high protein diet	Conventional diabetes diet	6 weeks	30% energy from carbohydrates (vs. 50% in control)	HbA1c reduction
Wang et al. [13]	2018	China	56	Low-carbohydrate diet	Low-fat diet	3 months	Not explicitly quantified	HbA1c reduction
Goday et al. [14]	2016	Spain	89	Very-low-calorie ketogenic diet	Low-calorie diet	4 months	<50 g/day	HbA1c reduction

Risk of Bias

The risk of bias for the included studies was assessed using the Cochrane RoB 2 tool, and the results are summarized in Table 2.

**Table 2:** Risk of bias assessment using the Cochrane Risk of Bias 2 tool

Study	Randomization	Deviations from intended interventions	Missing outcome data	Measurement of outcome	Selection of reported result	Overall risk
Saslow et al. [8]	Low risk	Some concerns	Some concerns	Low risk	Some concerns	Some concerns
Saslow et al. [9]	Low risk	Some concerns	Low risk	Low risk	Some concerns	Some concerns
Dorans et al. [10]	Low risk	Some concerns	Low risk	Low risk	Some concerns	Some concerns
Chen et al. [11]	Some concerns	Some concerns	Some concerns	Low risk	Some concerns	Some concerns
Thomsen et al. [12]	Low risk	Low risk	Low risk	Low risk	Some concerns	Some concerns
Wang et al. [13]	Some concerns	Some concerns	Some concerns	Low risk	Some concerns	Some concerns
Goday et al. [14]	Some concerns	Some concerns	Some concerns	Low risk	Some concerns	Some concerns

Primary Outcome: HbA1c

**Figure 2:**
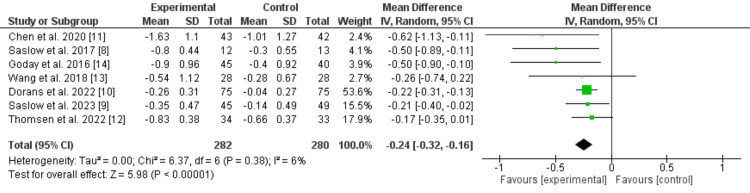
Forest plot showing the effect of low-carbohydrate diets on HbA1c

A pooled analysis using a random-effects model demonstrated that low-carbohydrate diets were associated with a statistically significant reduction in HbA1c compared to control diets: MD=-0.24% (95% CI: -0.32 to -0.16; p<0.00001). The pooled effect of low-carbohydrate diets on HbA1c is shown in Figure 2.

Heterogeneity

I² was 6%, and Chi² was 6.37 (p=0.38), indicating low heterogeneity and high consistency across studies.

Sensitivity Analysis

Sensitivity analysis demonstrated that removal of individual studies did not significantly alter the overall effect estimate, confirming the robustness of the findings.

Publication Bias

**Figure 3:**
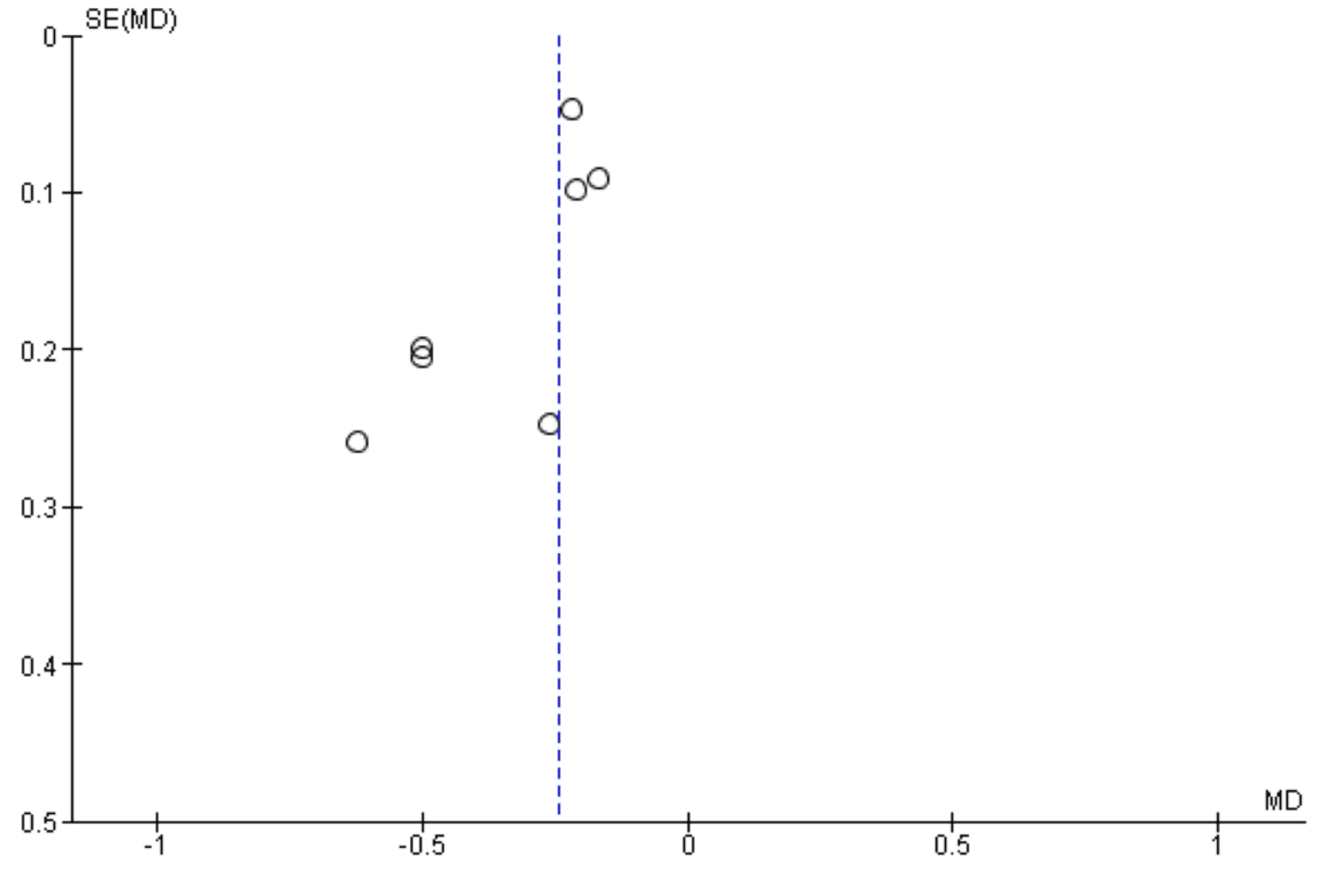
Funnel plot assessing publication bias

Publication bias was assessed using a funnel plot (Figure 3). Visual inspection suggested no clear evidence of major asymmetry. However, interpretation is limited due to the small number of included studies.

Discussion

This meta-analysis of seven RCTs demonstrates that low-carbohydrate diets are associated with a statistically significant reduction in HbA1c in individuals with T2DM. The pooled effect size (-0.24%) indicates a modest but meaningful improvement in glycemic control compared with control diets.

Interpretation of Findings

The observed reduction in HbA1c, although moderate in magnitude, is clinically relevant. Even small decreases in HbA1c have been associated with reductions in the risk of diabetes-related complications [2]. Importantly, this effect was achieved through dietary modification alone, highlighting the potential of low-carbohydrate diets as a non-pharmacological intervention in diabetes management [1,3].

The consistency of findings across studies is further supported by the low heterogeneity observed (I²=6%), as illustrated in the forest plot (Figure 2). This suggests that despite differences in study populations, durations, and specific dietary protocols, the overall effect of carbohydrate restriction on glycemic control is stable and reproducible.

Due to the limited number of included studies (n=7), subgroup analyses and meta-regression were not performed, which limits further exploration of potential sources of variability and may affect the interpretation of the pooled effect.

Comparison With Existing Literature

The findings of this analysis are consistent with previous systematic reviews and meta-analyses that have reported beneficial effects of low-carbohydrate diets on glycemic outcomes [5,7], as well as more recent analyses reporting similar short-term HbA1c reductions [15]. While some studies have demonstrated larger reductions in HbA1c, particularly with very-low-carbohydrate or ketogenic diets, the present analysis reflects a more conservative and realistic estimate across varying dietary intensities.

Recent evidence also suggests that the metabolic effects of low-carbohydrate diets may vary across populations and tend to diminish over time, highlighting the importance of long-term adherence and individualized dietary approaches [5,15].

This variability in effect size may be explained by differences in carbohydrate thresholds among included studies, ranging from moderate restriction (~130 g/day) to more intensive ketogenic approaches (<50 g/day). Such heterogeneity in dietary definitions is a common challenge in nutritional research and may influence the magnitude of observed effects. These findings are visually supported by the pool analysis (Figure 2).

Additionally, while low-carbohydrate diets may improve glycemic control, potential adverse effects should be considered, including increases in low-density lipoprotein cholesterol in some individuals, as well as possible micronutrient deficiencies and reduced dietary fiber intake.

Potential Mechanisms

Several physiological mechanisms may explain the observed benefits of low-carbohydrate diets. Reduction in carbohydrate intake leads to decreased postprandial glucose excursions and lower insulin demand [4]. Additionally, low-carbohydrate diets may improve insulin sensitivity and promote weight loss, both of which contribute to improved glycemic control [5].

Furthermore, limiting dietary carbohydrates reduces glycemic variability [1], which has been increasingly recognized as an important factor in diabetes management beyond HbA1c alone.

Strengths

This study has several strengths. First, only RCTs were included, enhancing the overall quality of evidence. Second, the use of a random-effects model accounts for potential variability across studies. Third, the low heterogeneity observed supports the reliability and consistency of the findings.

A structured risk of bias assessment using the Cochrane RoB 2 tool was conducted for all included studies (Table 2), allowing for a transparent and systematic evaluation of study quality and enhancing the methodological rigor of this meta-analysis.

Additionally, sensitivity analysis confirmed that the results were robust and not driven by any single study, further strengthening the validity of the conclusions.

Limitations

Several limitations should be acknowledged. The number of included studies was relatively small, which limits the power to detect publication bias and reduces the generalizability of findings. Additionally, variation in dietary protocols, including differences in carbohydrate thresholds and intervention duration, may influence outcomes.

Some studies required transformation of reported data (e.g., conversion from confidence intervals to standard deviations), which may introduce minor estimation error. Furthermore, differences in adherence to dietary interventions were not consistently reported and could affect the magnitude of the observed effect.

A structured risk of bias assessment using the Cochrane RoB 2 tool indicated that most included studies had some concerns overall rather than uniformly low risk of bias. The primary limitations were related to deviations from intended interventions, due to the open-label nature of dietary trials, and to missing outcome data in studies with longer follow-up durations. These factors may reduce confidence in the pooled effect estimate and could potentially lead to the overestimation of treatment effects. Therefore, the findings of this meta-analysis should be interpreted with appropriate caution despite the low statistical heterogeneity observed.

The definition of low-carbohydrate diet varied substantially across the included studies, ranging from very-low-carbohydrate ketogenic interventions (≤30-50 g/day) to more moderate carbohydrate restriction (up to 130 g/day). This heterogeneity may have masked differential effects between ketogenic and moderate low-carbohydrate approaches.

Due to the limited number of included studies (n=7), subgroup analyses and meta-regression were not performed. As a result, potential sources of variability, including differences in intervention intensity, duration, and participant characteristics, could not be formally explored.

This systematic review and meta-analysis was conducted by a single author, which represents a methodological limitation. Although study selection, full-text assessment, and data extraction were performed using predefined criteria with repeated verification, the absence of independent dual review increases the potential risk of selection bias and data extraction errors compared to reviews involving multiple reviewers.

This study was not prospectively registered in a database such as PROSPERO, which may increase the risk of reporting bias and limit methodological transparency.

Two of the seven included studies enrolled mixed populations, including participants with prediabetes, which may limit the direct applicability of the findings specifically to individuals with established T2DM.

Clinical Implications

The findings of this meta-analysis support the use of low-carbohydrate diets as an effective dietary strategy for improving glycemic control in individuals with T2DM. While the magnitude of HbA1c reduction is modest, it is clinically meaningful and may complement pharmacological therapies [1,3].

Clinicians may consider low-carbohydrate diets as part of individualized treatment plans, taking into account patient preferences, adherence potential, and overall nutritional balance.

## Conclusions

Low-carbohydrate diets are associated with a statistically significant and clinically relevant reduction in HbA1c in patients with T2DM. These findings support their role as a viable dietary approach in diabetes management, although further large-scale and long-term studies are warranted to confirm these results and optimize dietary recommendations.

## Appendices

Search strategy

Date of search: April 30, 2026

Date range: From database inception to March 2026

PubMed (MEDLINE)

Search string: ("low-carbohydrate diet"[Title/Abstract] OR "low carb diet"[Title/Abstract] OR "low-carbohydrate diets"[Title/Abstract] OR "very low carbohydrate diet"[Title/Abstract] OR "very low-carb diet"[Title/Abstract] OR "ketogenic diet"[Title/Abstract] OR "keto diet"[Title/Abstract] OR "very low-calorie ketogenic diet"[Title/Abstract]) AND ("Diabetes Mellitus, Type 2"[MeSH Terms] OR "type 2 diabetes"[Title/Abstract] OR "T2DM"[Title/Abstract] OR "type II diabetes"[Title/Abstract]) AND ("Glycated Hemoglobin A"[MeSH Terms] OR "HbA1c"[Title/Abstract] OR "hemoglobin A1c"[Title/Abstract] OR "glycated hemoglobin"[Title/Abstract] OR "glycosylated hemoglobin"[Title/Abstract])

Study design filter applied: Randomized controlled trials

Number of records retrieved: 47

Cochrane Library

Search string: ("low-carbohydrate diet" OR "low carb diet" OR "ketogenic diet" OR "keto diet" OR "very low carbohydrate diet" OR "very low-calorie ketogenic diet") AND ("type 2 diabetes" OR T2DM) AND (HbA1c OR "glycated hemoglobin" OR "glycated haemoglobin")

Number of records retrieved: 161

Google Scholar (Supplementary Search)

Search string: ("low-carbohydrate diet" OR "ketogenic diet") AND ("type 2 diabetes" OR T2DM) AND (HbA1c OR "glycated hemoglobin")

Google Scholar was used as a supplementary source to identify potentially relevant studies.

The first 200 results, sorted by relevance, were screened.

Due to limited reproducibility, these results were not included in the formal PRISMA flow diagram.

Additional Sources

Reference lists of included studies and relevant systematic reviews were hand-searched for additional eligible randomized controlled trials.

Search Process

All searches and screening procedures were performed by a single reviewer.
